# Physical activity and the risk of hip fracture in the elderly: a prospective cohort study

**DOI:** 10.1007/s10654-017-0312-5

**Published:** 2017-09-22

**Authors:** Ylva Trolle Lagerros, Essi Hantikainen, Karl Michaëlsson, Weimin Ye, Hans-Olov Adami, Rino Bellocco

**Affiliations:** 1 Department of Medicine, Clinical Epidemiology Unit T2, Karolinska University Hospital, 171 76 Stockholm, Sweden; 2 Department of Medicine, Clinic of Endocrinology, Metabolism and Diabetes, Karolinska University Hospital Huddinge, C2:84, 141 86 Stockholm, Sweden; 3 Department of Statistics and Quantitative Methods, University of Milano-Bicocca, Edificio U7, Via Bicocca degli Arcimboldi 8, 20126 Milan, Italy; 4 Department of Surgical Sciences, Section of Orthopedics, Uppsala Clinical Research Center, Akademiska sjukhuset ing. 61 6 tr, 751 85 Uppsala, Sweden; 5 Department of Medical Epidemiology and Biostatistics, Karolinska Institutet, PO Box 281, 171 77 Stockholm, Sweden; 6 Department of Epidemiology, Harvard University T H Chan School of Public Health, 677 Huntington Avenue, Boston, MA 02115 USA; 7 Clinical Effectiveness Research Group, Institute of Health and Society, University of Oslo, Sognsvannsveien 21, 0372 Oslo, Norway

**Keywords:** Epidemiology, Exercise, Hip fractures, Risk factors

## Abstract

Physical activity has been inversely associated with the risk of hip fracture, however, few studies have been conducted on the contributions from different domains of physical activity. This study was performed to investigate the association between daily household activities, leisure time physical activity, work-related physical activity and total physical activity during a 24-h period, and the risk of hip fracture. In the Swedish National March Cohort we followed 23,881 men and women aged of 50 and over from 1997 until 2010. Information on domain-specific physical activity was collected at baseline using a questionnaire. We fitted separate multivariable adjusted Cox proportional hazard models to each domain to obtain hazard ratios (HRs) with 95% confidence intervals (CIs). Each model was mutually adjusted for the other domains of physical activity. During a mean follow-up period of 12.2 years we identified 824 incidents of hip fracture. Subjects who spent less than 1 h per week engaged in daily household activities had an 85% higher risk of hip fracture than subjects spending ≥6 h per week carrying out daily household activities (HR 1.85; 95% CI 1.01–3.38). Subjects engaged in leisure time physical activities for >3.1 MET-h/day had a 24% lower risk of hip fracture (HR 0.76; 95% CI 0.59–0.98) than subjects spending <1.1 MET-h/day performing such activities. No association was found between hip fracture and work-related or total physical activity. We conclude that daily household activities and leisure time physical activity may independently decrease the risk of hip fracture in those aged 50 and over.

**Electronic supplementary material:**

The online version of this article (doi:10.1007/s10654-017-0312-5) contains supplementary material, which is available to authorized users.

## Introduction

Hip fracture is a major cause of hospitalization, impaired functional status and premature death among older adults. High-risk populations in Europe and North America have exhibited an increase in hip fracture incidence during the past 50 years, making hip fractures a serious public health issue [1]. Concomitantly, enormous changes have taken place in industrialized countries, where labor-intensive work has been replace by automation, and daily life generally requires less physical effort. An increasing proportion of the population has a sedentary lifestyle [2], which may lead to an increase in the incidence of hip fracture [3]. Regular weight-bearing exercise has an osteogenic effect [4–6], and it has been shown that athletes have both a more robust bone structure and improved strength of the femoral neck than non-athletes [7, 8]. In clinical trials, physical activity has been found to reduce falls and fractures through greater muscular strength and improved balance [9–13].

An inverse association has been found between physical activity and the risk of hip fracture in several observational cohort studies focusing on exercise and leisure time physical activity [14–16]. However, the relative contributions of different domains of physical activity, i.e., daily household activities, leisure time physical activity, work-related physical activity and total physical activity, to the reduction in the risk of hip fracture have not been widely studied. We therefore investigated the possible associations between these different domains of physical activity and the risk of hip fracture in a large prospective study, with a more comprehensive assessment of physical activity than in most previous epidemiological studies.

## Subjects and methods

The Swedish National March Cohort was established in conjunction with a four-day nation-wide fundraising activity organized by the Swedish Cancer Society in September 1997. All those participating in this event were invited to fill in a 36-page questionnaire concerning their physical activity and other lifestyle-related factors, as described previously [17]. Information on hip fractures, death or emigration during the follow-up period, was obtained from other national, continuously updated registers [18]. Accurate linkages between various registers—and thus essentially complete follow-up—were possible due to the use of individually unique personal identity numbers in Sweden which are assigned to all Swedish residents [19].

The total number of individuals who were given a questionnaire during the fund-raising event could not be assessed, but 43,880 participants handed in completed questionnaires. Participants who reported an incorrect personal identity number (n = 11), or who had died (n = 11) or emigrated (n = 491) before the start of the follow-up period were excluded. Furthermore, all subjects below the age of 50 (n = 19,761) were excluded as hip fracture is primarily a problem among the older population. The final study group thus consisted of 23,881 participants (9514 men and 14,367 women). Subjects for whom information was missing on daily household activities (n = 155, 0.7%), leisure time physical activity (n = 287, 1.2%), work-related physical activity (n = 433, 1.8%), or total physical activity (n = 2383, 10%) were excluded from the analyses.

All participants gave their informed consent to participate, thus giving their permission to access information on them from national registers. The Ethics Review Committee at Karolinska Institutet approved the study.

### Exposure information

All baseline information was self-reported by means of a questionnaire. Daily household activities were assessed in terms of the average number of hours (<1, 1–2, 3–4, 5–6, >6 h) spent per week on activities such as cleaning the house, working in the garden, as well as time spent walking and/or cycling to work.

Leisure time physical activity was assessed in the questionnaire by asking the subjects to report the number of hours per week they were engaged in sports, exercise and outdoor life activities including both summer and winter activities. The activities were divided into three intensity levels: light (e.g. casual walking), moderate (e.g. brisk walking, jogging or swimming), and heavy (e.g. competitive training or vigorous exercise). The reported number of hours was multiplied by the metabolic energy turnover (MET) for each intensity level (3 for light, 6 for moderate, 10 for vigorous). One MET corresponds to an energy expenditure of 3.5 ml O_2_ kg^−1^ min^−1^ or 1 kcal per kg body weight per hour [20]. These values were then converted to provide estimates of MET-h/day.

Work-related physical activity was determined with the question, “How physically demanding has your daily work/occupation been during the past 12 months?”. Participants were given the choices “Light, mostly sedentary”, “Light, some moving around”, “Rather strenuous”, and “Very strenuous”. If the participant selected either of the strenuous alternatives, they were asked to specify their activity according to the following: “Locomotion, such as walking, running, cycling, climbing or swimming”, or “Muscle power, such as lifting, bending, pushing, squeezing or twisting”, or “Other type of strain”.

To assess the total physical activity, participants also completed a question for the quantification of the total energy output associated with all physical activities during a typical 24-h period. This assessment had nine fixed categories representing 0.9, 1, 1.5, 2, 3, 4, 5, 6 and 8 METs. Participants were instructed to report the time spent at each intensity level during an average day and night. This provided a measure of their total physical activity, allowing the total MET-h/day to be estimated.

### Follow-up

Follow-up started on October 1st, 1997 and ended on December 31st, 2010, the date of hospitalization for hip fracture, emigration or death, whichever came first. Information on the participants was obtained from the Swedish National Inpatient Register, regarding information on hospital discharge diagnoses, the Swedish Population Register, regarding information on emigration, and the Swedish Cause of Death Register. We defined cases as those subjects who had experienced a hip fracture during the follow-up period, according to the International Coding of Disease (ICD) (ICD-10 codes S720, S721 or S722).

### Statistical analyses

The distribution of continuous and categorical potential confounders was reported by the level of daily household physical activities. Separate Cox proportional hazards regression models, with age as the underlying time scale, were used to estimate hazard ratios (HRs) and 95% confidence intervals (CIs) for each domain of physical activity. The distributions of leisure time and total physical activity were categorized into quartiles.

The multivariable models were adjusted for the following potential confounders: sex, level of education (7–9, 10–13 or >13 years of education, other); cigarette smoking (never, former, current); body mass index (BMI, kg/m^2^); diabetes (yes or no, self-reported), and osteoarthritis diagnosed before the beginning of follow-up (yes or no) based on the National Inpatient Register. Information on dietary calcium (mg/day) and vitamin D intake (μg/day), was derived from a semi-quantitative Food Frequency Questionnaire (FFQ) included in the baseline questionnaire and adjusted for energy intake [21]. We also adjusted the models for the Charlson comorbidity index [22] based on the National Inpatient Register by categorizing it into scores of 0, 1 or >1 to provide sufficient numbers of subjects in each category. Finally, each model was mutually adjusted for the remaining domains of physical activity to investigate domain-specific associations with the risk of hip fracture.

The proportional hazards assumption was tested using scaled Schoenfeld residuals. Stratified Cox regression models were fitted if the assumption of proportional hazards was violated in the multivariable model. Linear trends for daily household activities and work-related physical activity were investigated by including the values of the categories as continuous variables in the models. For leisure time and total physical activity the median value of each quartile was used as a continuous variable, and we fitted restricted cubic splines with knots at the 5th, 35th, 65th, and 95th percentiles of the particular exposure variable [23].

We also assessed potential effect modification by age (<65, 65–70, >70 years) and sex on the multiplicative scale [24] by testing the coefficient of the product term based on the likelihood ratio test comparing nested models. Similarly, we investigated potential interactions between the domains of physical activity.

In the first sensitivity analysis we excluded subjects with hip fractures due to traffic accidents, identified by the code for external cause of injury in the National Inpatient Register. However, this can be questioned as there are indications of comparable increases in the risks of low- and high-impact trauma fractures in association with decreasing bone density in the elderly [25, 26]. As stroke could be associated with hip fracture [27], the analysis was repeated excluding participants who had had a stroke before their hip fracture, based on the National Inpatient Register. We also repeated the analysis excluding subjects with a previous hip fracture (n = 25) and with any fracture before the beginning of the follow-up (n = 250).

Calcium and vitamin D supplements are common in the prevention of osteoporosis [28]. We therefore further adjusted each model for self-reported vitamin and mineral supplement use (yes, no), and investigated potential effect modification by supplement use. Moreover, hypertension and antihypertensive medication may have an impact on osteoporosis and the risk of fracture [29, 30]. We therefore also adjusted each model for self-reported antihypertensive medication, which was captured in our questionnaire by asking subjects whether they had ever been treated by a doctor for the disease.

The proportion of missing data regarding the confounding variables was 10% for cigarette smoking, 5% for BMI, 1.3% for educational level and less than 1% for diabetes, dietary calcium and D-vitamin intake. We therefore conducted supplementary analyses after imputing missing data for both exposure and confounders using the multiple imputation chained equation procedure [31].

All statistical analysis was performed with Stata: Release 13 (Statistical Software, College Station, TX: StataCorp LP.) Reported probabilities (*p* values) were two-sided, and values less than 0.05 were considered to indicate statistically significant differences.

## Results

The baseline characteristics of all the subjects, divided according to level of daily household activities, are given in Table 1. The mean age at baseline was 63.1 years (SD 8.3), and the cohort consisted of about 60% women. During the mean follow-up period of 12.2 years there were 824 cases of hip fracture [274 (33.3%) males and 550 (66.7%) females] among the 23,881 participants, corresponding to 3.45% of the total sample. Thirty-five of the hip fractures were due to suspected high-energy trauma accidents, and mean age at hip fracture was 80.1 years (SD 7.9 years). At the start of follow-up, 47% were retired. Subjects who were active more than, or equal to 6 h per week had a lower BMI, smoked to a lesser degree, had a higher dietary calcium intake, a higher consumption of vitamin and mineral supplements, were more likely to be retired, and spent more time engaged in leisure time physical activities. Diabetes and osteoarthritis were more common among those who were active less than 1 h per week.

**Table 1:** Selected baseline characteristics of the participants included in the study, categorized by daily household activity level (for example, gardening, household activities and commuting hours per week)

Variable			Daily household activities (h/week)			
		<1	1–2	3–4	5–6	≥6
Number of participants	Total 23,881	356	2653	6610	5395	8712
Age (years, mean, SD)	63.1 (8.3)	62.2 (9.3)	61.7 (8.5)	62.3 (8.4)	63.4 (8.4)	64.0 (8.0)
Gender (% men)	39.8	53.9	47.3	39.3	35.9	39.8
Height (cm), mean (SD)	169.5 (8.7)	171.5 (9.3)	170.8 (9.0)	169.6 (8.8)	169.0 (8.5)	169.3 (8.7)
Weight (kg), mean (SD)	72.4 (12.1)	78.3 (13.7)	75.5 (13.0)	73.1 (12.0)	71.4 (11.7)	71.3 (11.8)
Body mass index (kg/m^2^), mean (SD)	25.1 (3.4)	26.5 (3.9)	25.8 (3.8)	25.3 (3.4)	24.9 (3.2)	24.8 (3.2)
Waist circumference (cm), mean (SD)	86.9 (11.8)	93.3 (12.4)	89.9 (12.4)	87.5 (11.8)	86.1 (11.6)	86.0 (11.4)
Current smoking (%)	5.3	10.6	7.8	6.1	4.7	5.7
Alcohol (g/month), mean (SD)	295.2 (592.2)	310.2 (428.8)	357.7 (1360.9)	299.2 (399.6)	274.7 (367.4)	287.0 (426.8)
Education (≥13 years, %)	29.3	32.3	34.3	32.4	31.3	25.2
Energy intake (kJ/day), mean (SD)	8784.3 (2661.8)	8299.3 (2748.8)	8469.9 (2764.6)	8571.3 (2622.6)	8779.6 (2507.9)	9079.5 (2713.4)
Calcium intake (mg/day), mean (SD)	1200.2 (460.6)	1095.6 (487.4)	1143.1 (488.3)	1169.3 (444.2)	1210.5 (448.9)	1239.6 (464.5)
Hormonal replacement therapy^a^ (%)	43.4	45.3	45.3	46.9	45.3	42.8
Vitamin D intake (μg/day), mean (SD)	4.3 (2.2)	4.1 (2.2)	4.1 (2.4)	4.2 (2.2)	4.4 (2.1)	4.5 (2.3)
Diabetes (%)	3.5	6.8	5.0	3.8	3.2	2.9
Charlson comorbidity index, mean (SD)	0.3 (0.8)	0.6 (1.3)	0.3 (0.9)	0.3 (0.8)	0.3 (0.8)	0.3 (0.8)
Osteoarthritis (%)	2.6	5.1	3.1	2.2	2.4	2.6
Leisure time physical activity (METh/day), mean (SD)	2.3 (1.9)	0.8 (1.2)	1.3 (1.3)	1.8 (1.4)	2.3 (1.6)	3.1 (2.3)

The Swedish National March Cohort

^a^Females only

The incidence rates and hazard ratios for hip fracture, according to hours of daily household activities, with the highest activity level as the reference category, are reported in Table 2. We found an inverse association between participating in daily household activities and hip fracture, with the highest HRs in subjects spending less than 1 h per week on daily household activities (HR 2.13 (CI 95% 1.32–3.43) compared to subjects in the highest activity level (i.e. ≥6 h a week)). After adjusting for potential confounders, the multivariate-adjusted HRs associated with low levels of physical activity were 1.34 (95% CI 1.01–1.78) for 1–2 h of daily household activities per week and 1.85 (95% CI 1.01–3.38) for less than 1 h per week, compared to subjects engaging in the highest level of daily household activities, with a significant trend (*p* value = 0.03).

**Table 2:** Hip fracture incidence rates and hazard rates (HR), with 95% confidence intervals (CI) in brackets, for the effect of daily household activities (for example, gardening, household activities and commuting, hours per week) on hip fracture among men and women in the Swedish National March Cohort

			Daily household activities (h/week)		
	<1	1–2	3–4	5–6	≥6
Number of incident cases of hip fracture	18	95	207	188	302
Person-years	5732	32,200	80,744	66,027	106,983
Incidence rate^a^	581.3	389.9	312.4	306.4	294.9
HR (95% CI)^b^	2.13 (1.32–3.43)	1.35 (1.07–1.70)	1.04 (0.87–1.25)	1.01 (0.84–1.21)	1.00 (reference)
HR (95% CI)^c^	1.85 (1.03–3.32)	1.39 (1.07–1.82)	1.08 (0.89–1.32)	0.90 (0.73–1.11)	1.00 (reference)
HR (95% CI)^d^	1.85 (1.01–3.38)	1.34 (1.01–1.78)	1.07 (0.87–1.32)	0.86 (0.69–1.07)	1.00 (reference)

^a^Incidence rates are adjusted for age and presented per 100,000 person-years

^b^Adjusted for age at enrollment and gender

^c^Adjusted for age at enrollment, gender, BMI, educational level, cigarette smoking status, calcium and D-vitamin intake, diabetes, osteoarthritis and Charlson comorbidity index

^d^Model c additionally adjusted for leisure time physical activity and work-related physical activity

After performing sensitivity analysis by excluding fractures due to traffic accidents (n = 35), the HR increased slightly to 1.95 (95% CI 1.07–3.56) for subjects who spent less than 1 h per week on daily household activities, compared to those spending ≥6 h per week. Excluding subjects with a diagnosis of stroke (n = 2107) before the hip fracture increased the HRs for subjects devoting less than 1 h a week to daily household activities to 2.06 (95% CI 1.10–3.86), compared to the most active participants. The HR for subjects spending 1–2 h on daily household activities decreased to 1.28 (95% CI 0.94–1.74).

**Fig. 1:**
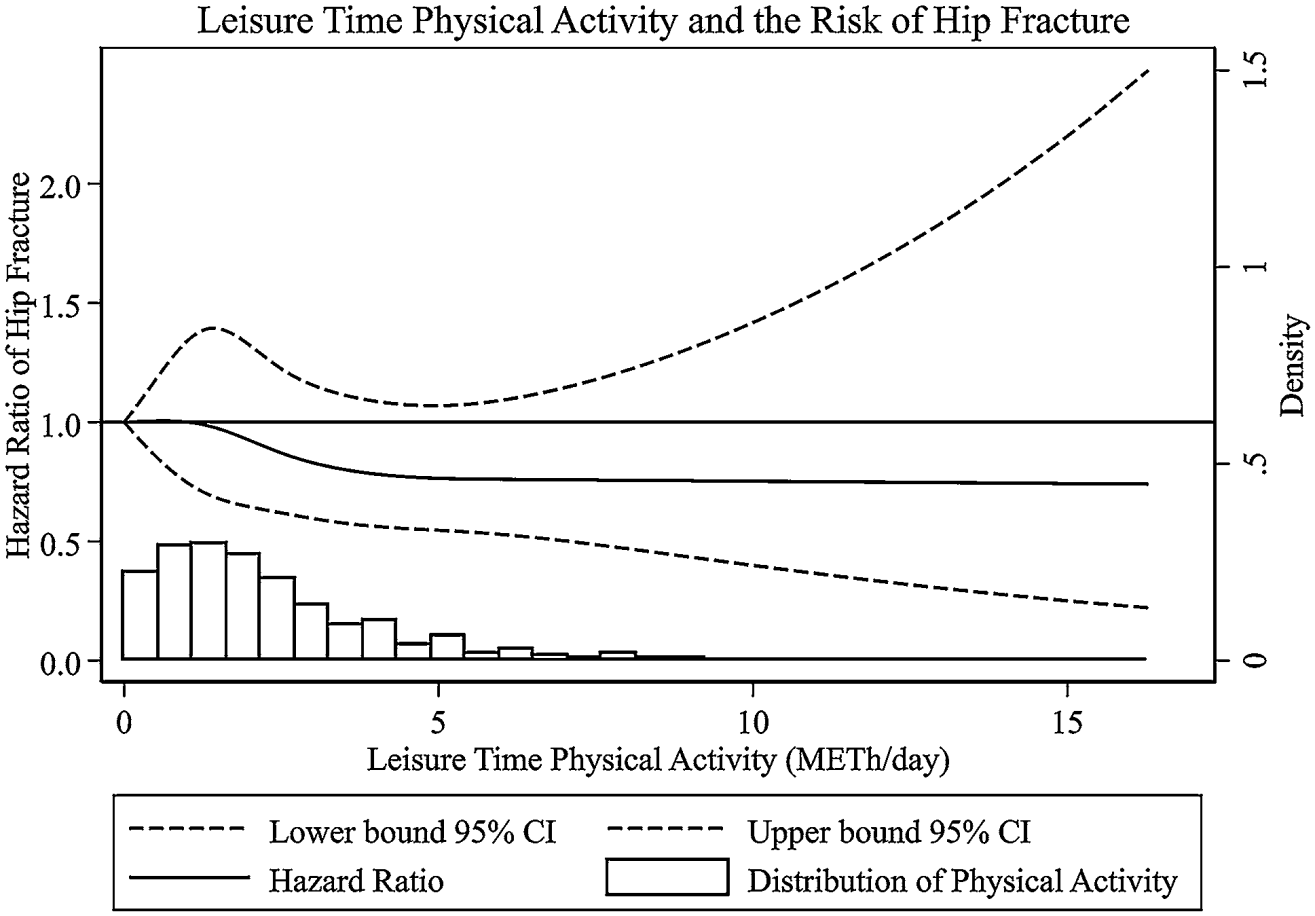
Multivariable-adjusted restricted cubic spline curve for the relation between leisure time physical activity (continuous, MET-h/day) and the risk of hip fracture. Adjustments were made for age, sex, BMI (kg/m^2^), educational level (7–9, 10–13 or >13 years of education, other), cigarette smoking (never, former, current), dietary calcium (mg/day) and D-vitamin intake (μg/day), diabetes (yes, no), osteoarthritis (yes, no), Charlson comorbidity index (0, 1 or >1), daily household activities (categorical) and work-related physical activity (categorical)

An inverse association was also found between leisure time physical activity and hip fracture, with the lowest HR in the third quartile, 1.8–3.1 MET-h/day (HR 0.75; 95% CI 0.62–0.91), compared to the first (<1.1 MET-h/day), and similar values in the fourth quartile (>3.1 MET-h/day) (HR 0.76; 95% CI 0.62–0.93). When adjusting the model for other confounders, the association remained significant for the fourth quartile, with a 24% lower hazard ratio compared to the first (HR 0.76; 95% CI 0.59–0.98) and a significant trend (*p* value = 0.03) (Table 3). The spline regression analysis did not reveal any departure from linearity (*p* value = 0.57) (Fig. 1). After excluding fractures caused by traffic accidents, the HR and CI remained almost similar, although non-significant, with a HR of 0.78 (95% CI 0.60–1.01) among those in the highest quartile of physical activity, compared to those in the lowest. In addition, estimates for subjects in the highest quartile did not change after excluding those with a diagnosis of stroke before their hip fracture (HR 0.75; 95% CI 0.57–0.99).

**Table 3:** Hip fracture incidence rates and hazard rate (HR) with 95% confidence intervals (CI) for the effect of leisure time physical activity on hip fracture among men and women in the Swedish National March Cohort

	Quartiles of leisure time physical activity levels (METh/day)			
	<1.1	1.1–1.8	1.8–3.1	>3.1
Number of incidents of hip fracture	239	226	184	148
Person-years	71,052	74,122	73,683	69,654
Incidence rate^a^	330.2	280.5	244.6	238.0
HR (95% CI)^b^	1.00 (reference)	0.85 (0.71–1.02)	0.75 (0.62–0.91)	0.76 (0.62–0.93)
HR (95% CI)^c^	1.00 (reference)	0.86 (0.70–1.06)	0.75 (0.61–0.94)	0.70 (0.55–0.89)
HR (95% CI)^d^	1.00 (reference)	0.92 (0.74–1.15)	0.83 (0.65–1.04)	0.76 (0.59–0.98)

^a^Incidence rates are adjusted for age and presented per 100,000 person-years

^b^Adjusted for age at enrollment and gender

^c^Adjusted for age at enrollment, gender, BMI, educational level, cigarette smoking status, calcium and D-vitamin intake, diabetes, osteoarthritis and Charlson comorbidity index

^d^Model c additionally adjusted for total daily household activities and work-related physical activity

We found no significant association between work-related physical activity and the risk of hip fracture (Supplementary Material, Table 1), nor any association between total physical activity and hip fracture; all the estimated HRs fluctuating around one (Supplementary Material, Table 2). No clear trend was found in the estimated dose–response relationship for total physical activity using spline regression (*p* value = 0.26).

We did not detect any incidence rate ratio heterogeneity on the multiplicative scale for the statistical interaction between daily household activities, leisure time physical activity, work-related physical activity or total physical activity with age and sex (*p* values ranging from 0.07 to 0.84). In addition, no significant interactions were found between the different domains of physical activity (*p* values ranging from 0.29 to 0.59).

After excluding subjects with a previous hip fracture, HRs remained the same (data not shown). Excluding subjects with any type of fracture before the beginning of the follow-up period did not affect the estimates for leisure time physical activity (data not shown). For daily household activities the adjusted HR was similar for subjects spending 1–2 h on daily household activities per week, with a HR of 1.38 (95% CI 1.03–1.86), and somewhat lower but non-significant for subjects spending less than 1 h per week on daily household activities, with a HR of 1.66 (95% CI 0.83–3.30). However, after the exclusion of any fractures, the number of cases in the fully adjusted model for the subgroup being active less than 1 h per week was only 9, which may have been too small to detect any effect. After further adjustments of the models for the use of vitamin and mineral supplements, HRs remained the same (data not shown). In addition, we found no effect modification between any of the domains of physical activity and supplement use on the multiplicative scale (*p* values ranging from 0.47 to 0.86). Moreover, adjusting the models for self-reported antihypertensive medication did not affect the pattern of the effect, however, estimates did not reach statistical significance (data not shown). Furthermore, our results remained stable after carrying out the analysis on the multiple imputed data (data not shown).

## Discussion

In this large prospective cohort study, we found that daily activities such as cleaning, gardening and commuting, as well as leisure time physical activity such as sports, exercise and other outdoor activities, were inversely associated with the risk of hip fracture in men and women. However, no significant associations were found between the risk of hip fracture and work-related physical activity or total physical activity.

Epidemiological studies have convincingly shown that physical activity reduces the risk of hip fracture; most evidence arising from studies focusing on the effect of moderate to vigorous leisure time physical activity and exercise [32]. Our results for leisure time physical activity support these conclusions, showing that more active subjects were at lower risk of hip fracture than the least active subjects. However, few studies have investigated daily activities such as walking, housework, gardening and cycling, which are most prevalent among the older population [33, 34]. A prospective study of 9704 elderly North American women found that spending more than 9 h per week on heavy household chores reduced the risk of hip fracture by 22% compared to subjects with an activity duration below 5 h per week [35]. Two case–control studies conducted among British men and women support these findings [36, 37].

In our study, men and women spending less than 3 h per week on daily household activities were at higher risk of hip fracture than subjects who were active at least 6 h per week. Although the definitions of exposure vary between studies, findings support the hypothesis that participation in daily domestic and commuting activities may help to prevent hip fracture in the elderly. The intensity of such activities can vary from low to vigorous, and we were not able to consider this factor. However, any physical activity, whether conducted during leisure time, or as part of daily household activities, has a positive effect on musculoskeletal and neuromuscular function [38].

Work-related physical activity contributes significantly to total physical activity until the age of retirement (about 65) [39], and may therefore be an important determinant in the risk of fracture. However, we found no significant association between work-related physical activity and the risk of hip fracture. Findings from previous studies are inconsistent [15, 39–42]. In the Women’s Health Initiative Observational Study, a large multi-ethnic cohort of 93,676 post-menopausal women, the occupational physical demand of up to three jobs held was considered, but work-related physical demand was not found to have any effect on the risk of hip fracture [42].

Our study has several limitations. Physical activity and other lifestyle factors were only assessed once, at baseline. We were therefore not able to capture any changes in exposure or potential confounders during follow-up. In the main form of exposure studied, i.e. household tasks, gardening and commuting, we were not able to take into account the intensity of the activity. Furthermore, the frequencies and durations of physical activity in the different domains were self-reported, which could have led to misclassification of the exposure. Our study focused on the population aged 50 and over, and the risk of disease increases with age. Health status is a strong confounder, since healthy subjects may be more physically active than those with an illness [43]. Subjects may change patterns of physical activity after disease onset [44]. If such changes are related to the outcome, this could lead to misclassification and an over- or underestimation of the effect. However, we attempted to consider health status by adjusting our model for potential comorbidity based on the Charlson comorbidity index [22].

We adjusted our analyses for diabetes and osteoarthritis. Although information on osteoarthritis was obtained from national registers, both diabetes and osteoarthritis are known to be under diagnosed. Furthermore, there were few DXA machines available in Sweden in the mid-1990s. There were and is, still no screening program for osteoporosis in Sweden. Thus, some individuals would have had these diseases, although they would not have been diagnosed, which could have led to residual confounding.

The subjects were all invited to take part in the study during a fund-raising event. This may have caused a bias towards healthy volunteers. While population-based cohorts often face the problems of poor response rates and incomplete follow-up, the drawbacks of a non-representative sample must be weighed against the fact that choosing a restricted sample can enhance the feasibility of the study, and increase the prevalence of the exposure and completeness of the follow-up, all of which increase the validity and precision of the study [45]. For example, the level of missing data was unusually low in our study.

The strengths of our study are its prospective design, with a baseline questionnaire covering a number of potential confounding factors, the large sample size, as well as detailed information on different domains of physical activity based on a validated questionnaire. Our outcome is also well defined. Since patients with hip fracture are hospitalized, the follow-up of the incidence of hip fracture was practically completely captured through cross-linkage with the National Inpatient Register.

According to our findings, participation in leisure time physical activities or in daily household activities may, independently of each other, decrease the risk of hip fracture in the older population. This may be an important health message to the elderly population that continuing an active lifestyle is more important than taking part in organized exercise programs in sports or rehabilitation facilities. In addition, a strong determinant for physical inactivity in the elderly population is lack of interest [46]. Therefore, implementing low to moderate activities in a daily routine may be easier and more sustainable, preventing a lapse of physical activity and its consequences later in life.

## Electronic supplementary material

Below is the link to the electronic supplementary material.

Supplementary material 1 (DOCX 22 kb)
