# Supplementary material for: Physical activity and the risk of hip fracture in the elderly: a prospective cohort study
Source: Eur J Epidemiol. 2017 Sep 22;32(11):983–91. doi: 10.1007/s10654-017-0312-5 (PMC5684287; doi:10.1007/s10654-017-0312-5)
Supplement: Supplementary file 1 — Supplementary material 1 (DOCX 22 kb) [file 10654_2017_312_MOESM1_ESM.docx]

**Article Title:** Physical Activity and the risk of Hip Fracture in the Elderly – A Prospective Cohort Study

**Journal Name:** European Journal of Epidemiology

**Author Names:**

Ylva Trolle Lagerros*, MD, PhD1,2, Essi Hantikainen*, MSc3, Karl Michaëlsson, MD, PhD4, Weimin Ye, MD, PhD5, Hans-Olov Adami, MD, PhD5,6,7, Rino Bellocco, PhD3,5

^1^ Department of Medicine, Clinical Epidemiology Unit T2, Karolinska University Hospital, SE-171 76 Stockholm, Sweden

^2^ Department of Medicine, Clinic of Endocrinology, Metabolism and Diabetes, Karolinska University Hospital Huddinge, C2:84, 141 86 Stockholm, SE-Sweden

^3^ Department of Statistics and Quantitative Methods, University of Milano-Bicocca, Edificio U7, Via Bicocca degli Arcimboldi 8, 20126 Milan, Italy

^4^ Department of Surgical Sciences, Section of Orthopedics, Uppsala Clinical Research Center, Akademiska sjukhuset ing. 61 6 tr, 751 85 Uppsala, SE-Sweden

^5^ Department of Medical Epidemiology and Biostatistics, Karolinska Institutet, PO Box 281, SE-171 77 Stockholm, Sweden

^6^ Department of Epidemiology, Harvard University T H Chan School of Public Health, 677 Huntington Avenue, Boston, MA 02115, USA

^7^ Clinical Effectiveness Research Group, Institute of Health and Society, University of Oslo, Sognsvannsveien 21, 0372, Oslo, Norway

**Corresponding Author:** Ylva Trolle Lagerros, MD, PhD, E-mail: [ylva.trolle@ki.se](mailto:ylva.trolle@ki.se)

Supplementary Material Table 1. Hip fracture incidence rates and hazard ratios (HR) with 95% confidence intervals (CI) in brackets for the effect of work-related physical activity on the risk of hip fracture among men and women in the Swedish National March Cohort.

|  |  | | **Physical activity level at work** | | | |  | |
| --- | --- | --- | --- | --- | --- | --- | --- | --- |
|  | **Light, mostly sitting** | **Light, some**  **moving around** | | **Strenuous locomotion** | **Strenuous muscular and other activity** | **Strenuous muscular and locomotion** | | ***p for trend*** |
| Number of incident cases of hip fracture | 52 | 603 | | 74 | 36 | 19 | |  |
| Person-years | 36,262 | 192,491 | | 27,040 | 21,304 | 9,887 | |  |
| ^a^ Incidence rate | 328.6 | 309.4 | | 303.3 | 246.6 | 352.1 | |  |
| ^b^ HR (95% CI) | 1.00 (reference) | 0.87 (0.65-1.17) | | 0.88 (0.61-1.26) | 0.72 (0.47-1.11) | 1.09 (0.65-1.85) | | 0.60 |
| ^c^ HR(95% CI) | 1.00 (reference) | 1.08 (0.76-1.55) | | 1.09 (0.70-1.69) | 1.00 (0.61-1.65) | 1.75 (0.97-3.17) | | 0.32 |
| ^a^ Incidence rates are adjusted by age and presented per 100,000 person-years.  ^b^ Adjusted for age at baseline and sex.  ^c^ Adjusted for age at baseline, sex, BMI (kg/m^2^), educational level (7-9, 10-13 or >13 years, other), cigarette smoking status (never, former, current), calcium (mg/day) and D-vitamin intake (mcg/day), diabetes (yes, no), osteoarthritis (yes, no), Charlson comorbidity index (0, 1 or >1), leisure time physical activity (METh/day) and daily household activity (categorical). MET=Metabolic Energy Turnover | | | | | | | | |

Supplementary Material Table 2. Hip fracture incidence rates and hazard ratios (HR) with 95% confidence intervals (CI) in brackets for the effect of total physical activity on the risk of hip fracture among men and women in the Swedish National March Cohort.

| **Total physical activity level (METh/week)** | | | | | |
| --- | --- | --- | --- | --- | --- |
|  | <30.8 | 30.8-35.4 | 35.4-44.4 | >44.4 | ***p for trend*** |
| Number of incident cases of hip fracture | 226 | 142 | 139 | 165 |  |
| Person-years | 64,576 | 66,752 | 66,647 | 66,015 |  |
| ^a^ Incidence rate | 345.5 | 288.3 | 254.9 | 277.0 |  |
| ^b^ HR (95% CI) | 1.00 (reference) | 0.89 (0.72-1.10) | 0.80 (0.65-1.00) | 0.95 (0.77-1.17) | 0.49 |
| ^c^ HR (95% CI) | 1.00 (reference) | 0.93 (0.73-1.17) | 0.81 (0.64-1.03) | 0.92 (0.73-1.16) | 0.87 |
| ^a^ Incidence rates are adjusted by age and presented per 100,000 person-years.  ^b^ Adjusted for age at baseline and sex.  ^c^ Adjusted for age at baseline, sex, BMI (kg/m^2^), educational level (7-9, 10-13 or >13 years, other), cigarette smoking status (never, former, current), calcium (mg/day) and D-vitamin intake (mcg/day), diabetes (yes, no), osteoarthritis (yes, no) and Charlson comorbidity index (0, 1 or >1). | | | | | |
